# In Vitro Study of Comparative Evaluation of Marginal and Internal Fit between Heat-Pressed and CAD-CAM Monolithic Glass-Ceramic Restorations after Thermal Aging

**DOI:** 10.3390/ma13194239

**Published:** 2020-09-23

**Authors:** Roxana-Diana Vasiliu, Sorin Daniel Porojan, Liliana Porojan

**Affiliations:** 1 Department of Dental Prostheses Technology (Dental Technology), University of Medicine and Pharmacy “Victor Babes”, Eftimie Murgu Sq. no. 2, 300041 Timisoara, Romania; roxana.vasiliu@umft.ro; 2 Department of Oral Rehabilitation (Dental Technology), University of Medicine and Pharmacy “Victor Babes”, Eftimie Murgu Sq. no. 2, 300041 Timisoara, Romania; porojan.sorin@umft.ro

**Keywords:** glass-ceramic, CAD/CAM, marginal and internal adaptability, replica technique, micro-CT evaluation

## Abstract

The accuracy of newly developed ceramic materials is still being studied. Marginal and internal adaptation are known factors that have an essential impact on the long term success of dental restorations. The aim of this in vitro study was to evaluate the marginal and internal fit of heat-pressed and milled monolithic glass-ceramic restorations based on their ceramic type, processing technique, and in vitro thermocycling. Thirty-two crowns were studied and divided into four groups (*n* = 8), according to the ceramic material (feldspathic glass-ceramic (F) and zirconia reinforced lithium silicate glass-ceramic (ZLS)) and to their technological obtaining processes (milling (M) and heat-pressing (P)). A typodont preparation was scanned with a D2000 3D scanner to obtain identical 32 resin 3D-printed abutment teeth. Marginal and internal gaps were measured using the silicone replica technique under 40× magnification. The crowns were further cemented and thermally aged for 10,000 cycles After cementation and thermocycling of the samples, marginal and internal gaps were assessed using micro-CT (micro-computed tomography)) analysis. Data were statistically analyzed using statistical tests. Significant differences were found before and after cementation and thermocycling among the tested materials (*p* < 0.05). Related to technological processing, significant differences were seen in the marginal area between FP and FM (*p* < 0.05) Significant differences were also found in the axial and occlusal areas between the ZLSP and ZLSM. Thermocycling and cementation did not have a significant effect on the tested materials (*p* < 0.05). The technological processes influenced the marginal and internal fit of the crowns in favor of the CAD/CAM (computer aided design/computer aided manufacturing)technologies. Thermal aging had little effect on marginal adaptability; it increased the values for all the tested samples in a small way, but the values remained in their clinically acceptable range for all of the crowns.

## 1. Introduction

The adaptability of the crowns, along with esthetics and fracture resistance, are essential factors in controlling the long-term success of restorations [1,2,3,4,5,6,7,8,9,10]. Excellent marginal adaptation influences percolation and plaque build-up by limiting them to a possible degree [11]. Crown adaptation is defined by the marginal and internal gaps [12]. The marginal gap term can be defined as the space between the crown and the preparation line. Holmes provides a significant definition of the term as “the discrepancy between the edge of the crown and the tooth’’ [13]. There can be inner gaps, defined as the space between the inner part of the restoration and the dental structure, while the discrepancy at the edge is named the marginal demarcation gap [14]. Usually, these gaps appear as a result of morphological disproportion between the tooth and the restoration. An increase in the marginal and internal gaps can result in cement dissolution, leading to microleakage caries and periodontal disease [15,16,17,18,19,20,21]. To assess the marginal gap, different methods were developed. Sorensen et al. classified these methods into four categories: direct observation; cross-sectional method, impression method, and using an explorer [22]. Impression techniques and visual examination must rely on the researcher’s experience, and this is often influenced by subjectivism [23]. Another factor that influences these techniques is the location of the demarcation line. It is harder to objectively evaluate the marginal gap when the demarcation line is subgingival [24,25]. The most widely used method is the replica technique, which consists in using a low viscosity silicone, but this can induce inaccurate results. The silicon impression can be damaged once removed from the die [26]. Another non-invasive method that provides useful information after cementing the samples is micro-CT analysis. Micro-CT is the perfect method to analyze the samples without the need for cutting them and has been used in dental research aside from other indications to assess the marginal and internal gaps in ceramic restorations [27,28,29].

This method permits the rotation of the investigated sample and the quantitative cementation areas. Over the years, many ceramic systems that may differ in chemical composition and technological processing have been developed. Two of the materials used clinically are feldspathic, and zirconia reinforced lithium silicate glass-ceramic. These materials can be milled from prefabricated blocks using the CAD/CAM technology systems or heat-pressed from prefabricated ingots. The CAD/CAM workflow brings numerous advantages in clinical practices compared to the conventional workflow. Some of this advantages are the comfort of the patient during scanning as well as reduced time and materials compared to a conventional impression.

Two methods can be used to process prefabricated CAD/CAM blocks. The first one, also known as hard milling, processes a fully sintered block to obtain the restorations and can lead to excessive tool wear and multiple defects [30]. The second developed method, green milling, utilizes only partially sintered materials. After the milling takes place, the restorations are fully sintered to dispose of the porosity [31]. An important aspect is that the software, the part of the CAD/CAM production, correlates the shrinkage of the prosthetic piece resulting after sintering. This aspect is directly linked to an appropriate marginal adaptation. The milled zirconia reinforced lithium silicate glass-ceramic (Vita Suprinity) is fabricated in pre-sintered blocks, which need additional crystallization. On the other hand, the milled feldspathic glass-ceramic (Vita Mark II) has the advantage of being milled in its final form, leading to fewer distortions attributed to additional firings. The heat-pressing method was developed in late-1980, enabling dental laboratories to obtain dental restorations using the lost-wax technique. The method uses fabricated ingots made of crystalline particles that are scattered in a glassy matrix. The obtained structure is very much alike that of the powder ceramics. Despite the structure, pressable ceramics provide restorations with less porosity, higher crystalline content, and great marginal adaptability. Ingots are made from heated powders until melting, and this composition is poured into a steel mold and left to cool. After the glass stage, the ingots are nucleated and crystalized in a specific heat-treatment. The sintering temperature depends on each processed ceramic and the final restoration is finished after staining [32,33].

Thermal aging is a popular method to accelerate the aging of ceramics by reproducing to some degree, the oral environment [34,35,36,37]. The method includes standardized thermal variations with baths ranging from 5 to 55 °C for several cycles. Thermal aging can predict the restoration’s longevity, and using it can simulate the behavior of the ceramic material [38].

The aim of this in vitro study was to evaluate the adaptability of monolithic glass-ceramic restorations in correlation to the type of ceramic, processing technique, and in vitro thermocycling.

The first null hypothesis was that the material type and processing technique would not affect the marginal adaptability of the monolithic heat-pressed and CAD-CAM ceramics. The second null hypothesis was that the thermal aging would not affect the marginal adaptability of the glass-ceramics.

## 2. Materials and Methods

### 2.1. Preparation Design

A typodont premolar tooth was prepared following:

(1) Margin design: 1 mm circumferential rounded chamfer;
(2) 6° convergence of axial wall;
(3) Axial reduction: 1.5 mm;
(4) Planar occlusal reduction of 1.5–2 mm; and
(5) Support lingual cusp beveled.

Thirty-two crowns were obtained and divided into four groups (*n* = 8), according to the ceramic material (feldspathic glass ceramic and zirconia reinforced lithium silicate glass-ceramic) and to their technological production (milling and heat-pressing).

To provide standardization for all the tested crowns, a typodont preparation was scanned with a D2000 3D scanner (3Shape, Copenhagen, Denmark) to obtain identical 32 resin 3D-printed abutment teeth (Freeprint Model 2.0, Detax GmbH & Co., Ettlingen, Germany).

### 2.2. CAD/CAM Manufacturing Procedure

**Figure 1:**
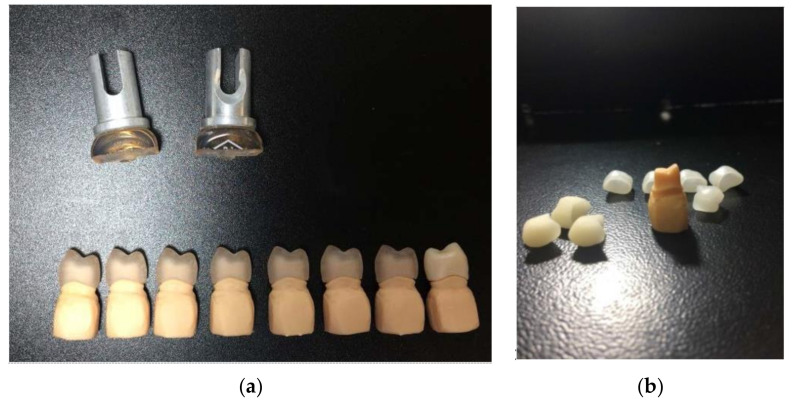
(**a**) Pre-crystalized ZLSM crowns (Vita Suprinity, Vita Zahnfabrick, Germany) and (**b**) 3D printed abutment and the milled wax patterns.

After scanning the resin abutments, and obtaining a proper design, sixteen crowns were milled. All-ceramic crowns were milled from zirconia reinforced lithium silicate glass-ceramic and feldspathic glass-ceramic blocks (Vita Suprinity; Vita Mark II, Vita Zahnfabrick, Bad Sackingen, Germany) (Table 1) (Figure 1). Crowns from ZLS blocks need an additional crystallization step after milling, according to the manufacturer’s instructions (Figure 1). Each crown was kept in the ceramic furnace at a final temperature of 850 °C for about 25 min to accomplish full crystallization. The crowns were glazed using the specific glaze for each ceramic (Figure 1).

### 2.3. Heat-Pressing Manufacturing Procedures

To provide standardization, 16 samples were milled from white Ceramill Wax (Amann Girrbach AG, Kobalch, Östereich) to be heat-pressed.

To obtain the heat-pressed ceramic samples, wax patterns were sprued and invested in a phosphate-bonded investment material (Bellavest SH; BEGO GmbH & Co. KG, Bremen, Germany).

**Figure 2:**
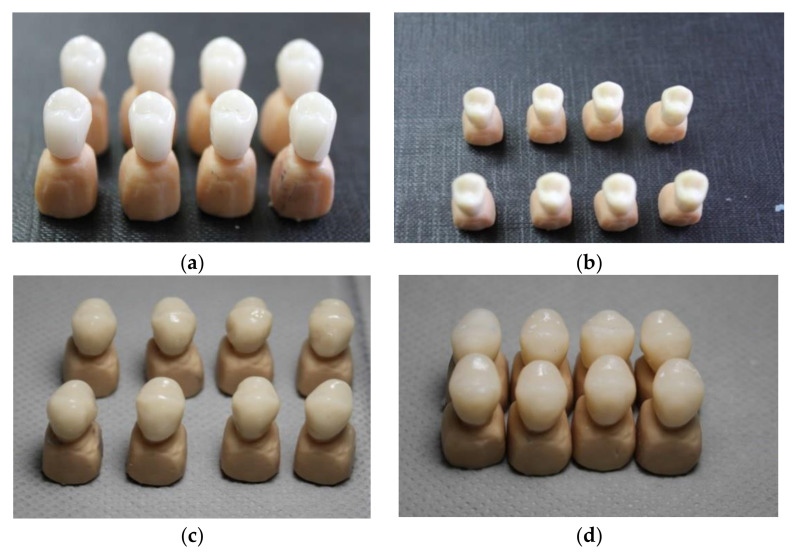
(**a**) Milled feldspathic glass ceramic, (**b**) Milled zirconia reinforced lithium silicate glass ceramic (**c**) Heat-pressed feldpsathic glass-ceramic (**d**) Heat-pressed zirconia reinforced lithium silicate glass-ceramic.

Molds were preheated at 900 °C for 60 min, and prefabricated ingots with medium translucency (MT) (Celtra Press, Dentsply, Degudent; Vita PM9, Vita Zahnfabrick, Bad Säckingen, Germany) Figure 1 were pressed into ceramic specimens using a press furnace (Multimat 2 Touch+ Press Dentsply; Salzburg, Osterreich), following the manufacturer’s guidelines (Table 2). The mold was allowed to cool down for several hours, and the specimens were carefully devested by sandblasting using glass powders (50 μm) at a pressure of 4 bar. The sprues were cut, and samples were prepared for glazing. The crowns were glazed using the specific glaze for each ceramic (Table 3) (Figure 2).

### 2.4. Replica Technique in Marginal Adaptation Technique

**Figure 3:**
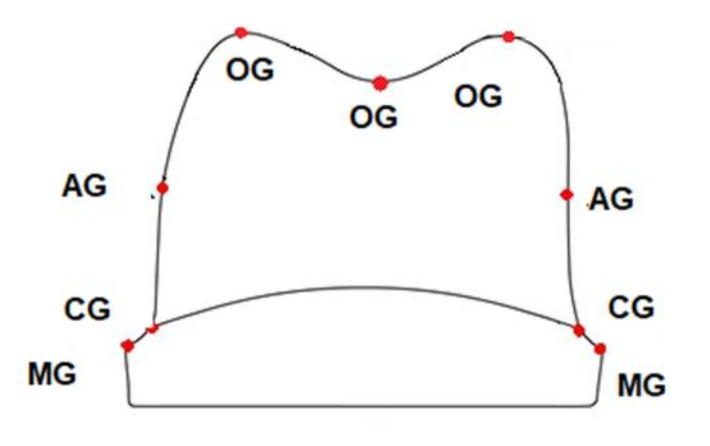
Anatomical distribution of the evaluation points for the replica technique and micro-CT measurements.

Before cementation, in order to assess the marginal gap, the impression technique was selected. The method involves using low and moderate viscosity material (Speedex, Coltene, Langenau, Germany). The crowns were filled up with light body silicone. After adapting the crown on the resin abutment, finger pressure was applied for about 15 s. When the polymerization completed, after three minutes, the crown was removed, and the light body silicon remained on the abutment. The medium body silicone was added and placed in the crowns on the light body impression material to provide support to the light body silicon for another three minutes. All registrations were made by the same person (Figure 3).

**Figure 4:**
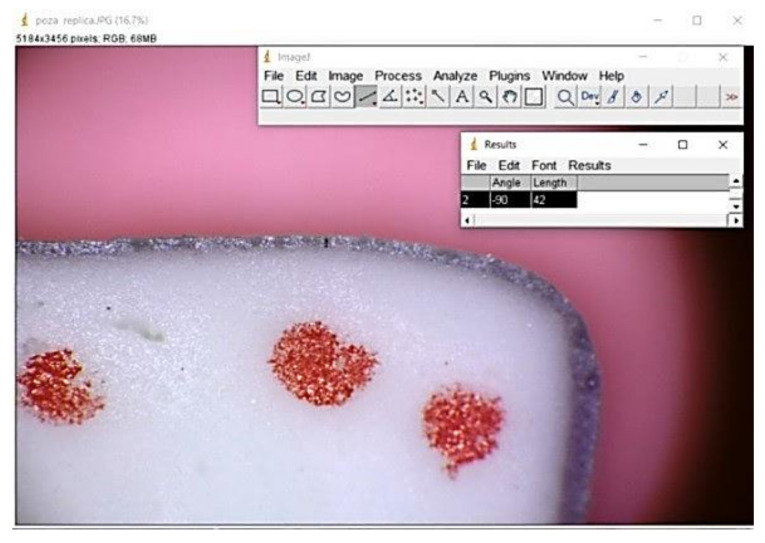
Replica technique with silicon in ImageJ.

The marginal and internal gaps were measured at nine points of each piece after cutting the completed silicon replica in the buccolingual directions. The measurements were made at a magnification of ×40 using the image microscope system (Leica DM 100; Mannheim, Germany) (Figure 4). The marginal adaptability was measured using image analysis software Image J (version 1.46, Java). Eighteen measuring locations were used (nine per section) to evaluate the thickness of the low viscosity silicon, corresponding to the cement space.

### 2.5. Cementation Procedures

All the evaluated crowns were cemented using a self-etch self-adhesive resin cement (Maxcem Elite, Self Etch, Kerr, CA, USA), following the producer’s instructions. The automixed cement was distributed on the inner surface of the ceramic crown, and the crown was carefully placed on the resin abutment with a finger pressure of 50 N. The cement excesses were cleaned using micro brushes. Light curing was applied using a UV-polymerization lamp, all carried out by the same person for 30 s on all the surfaces of the restorations (mesial, distal, lingual, and buccal).

### 2.6. Aging Process

Thermocycling was carried out for 10,000 cycles after cementing the samples on the resin abutments with a thermocycler (Thermocycler, SD Mechatronik, Feldkirchen-Westerham, Germany). Thermal cycles included the temperature of 5 °C and 55 °C and a dwelling time of 20 s between baths in distilled water.

### 2.7. Micro-CT Analysis

Median sections were made in the buccolingual direction for each specimen and evaluated using a micro-CT scanner (Nikon XT V 160, Minato, Tokyo, Japan), and the data were analyzed using the specific software MyVGL (Volume Graphics, Hidelberg, Germany). The evaluation was made on marginal and internal fit. A total of eighteen measuring locations (nine per section) were selected to evaluate the thickness of the cement. Measurements were made in µm and spread along with the entire preparation in order to assess correctly the marginal and internal fit of the ceramic crowns.

**Figure 5:**
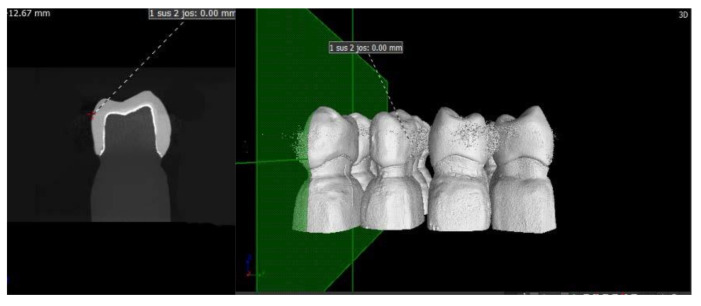
Images from the micro-CT program for the FP samples.

**Figure 6:**
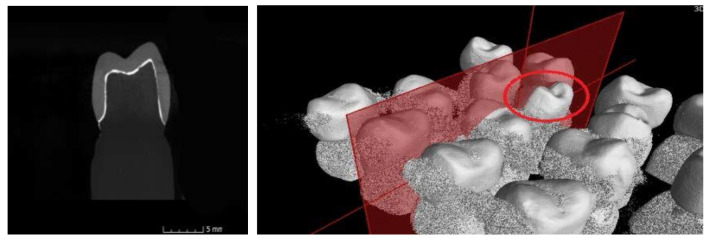
Images from the micro-CT program for the FM samples.

**Figure 7:**
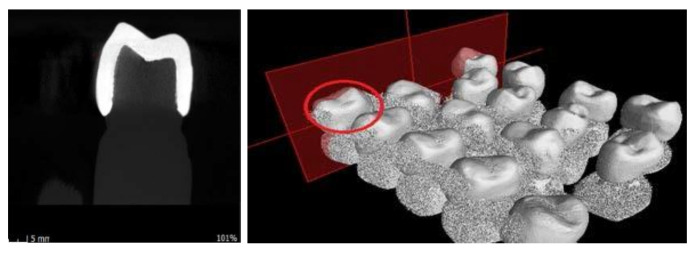
Images from the micro-CT program—ZLSP samples.

**Figure 8:**
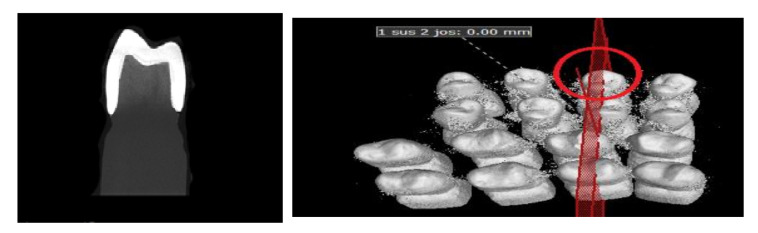
Images from the micro-CT program—ZLSM samples.

A scan was provided for each of the ceramic crowns cemented on the resin abutment using micro-CT and respecting the following conditions: before each scan calibration, image corrections were made to facilitate achieving the best contrast. For each ceramic specimen, a resolution of 4 µm per pixel was selected. The data were reconstructed with a resolution of 10 µm per voxel (Figure 5, Figure 6, Figure 7 and Figure 8).

### 2.8. Statistical Analysis

Collected data from the study were analyzed using two-way ANOVA tests for the marginal and internal fit before and after cementing and thermocycling using SPSS (IBM, Armonk, NY, USA). The *p* value for the tests was *p* < 0.05. 

## 3. Results

### 3.1. Before Cementation and Thermocycling

**Figure 9:**
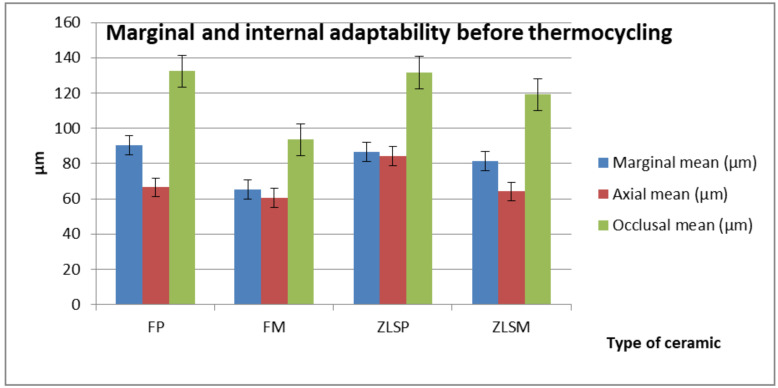
Mean values and standard deviation before cementing and thermocycling the ceramic crowns.

Statistical results are summarized in Table 4 and Table 5. Significant differences were found in the marginal, axial, and occlusal areas (*p* < 0.05) between the four groups of glass-ceramic (Figure 9).

Depending on the type of ceramic, significant differences were found in the marginal area between FM and ZLSP, where the FM glass-ceramic reported a better marginal fit than the ZLSP glass-ceramic. Furthermore, in the occlusal and axial areas area, significant differences were seen between the FP FM and ZLSPM. Significant differences in the occlusal areas were found between FP and ZLSM. The ZLSM glass-ceramic had a better occlusal fit with lower values compared to the FP glass-ceramic. Significant differences were seen in the marginal area between FPM and ZLSM. The ZLSM glass-ceramic reported lower marginal values, and therefore a better marginal fit compared to the FP glass-ceramic.

With regard to technological processing, significant differences were seen in the marginal area between FP and FM. The FM glass-ceramic reported lower values for the marginal area compared to the FP glass-ceramic.

Significant differences were also found in the axial and occlusal areas between the ZLSP and ZLSM. The best marginal and internal fit were seen in the FM crowns.

### 3.2. After Cementing and Thermocycling the Samples

**Figure 10:**
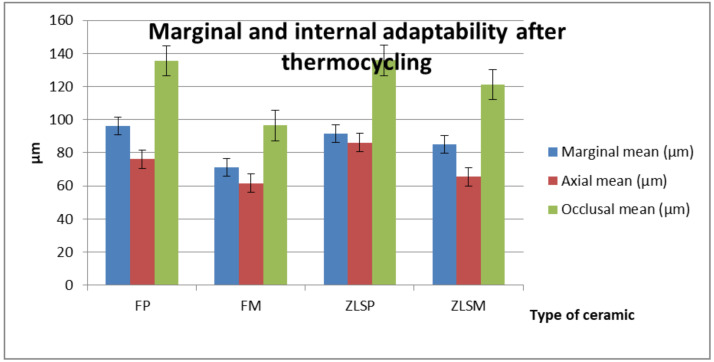
Mean values and standard deviation after cementing and thermocycling the ceramic crowns.

The micro-CT evaluation (Figure 10) of the ceramic samples showed significant differences between the studied groups, and the statistical results are reported in Table 6 and Table 7.

Depending on the type of glass-ceramic, significant differences were found in the marginal area between FM and ZLSP as well as between FM and ZLSM (*p* < 0.05). Thee FM glass-ceramic had lower values for the marginal fit compared to the other two ZLS glass-ceramics.

With regard to the processing technologies, in the marginal area, significant differences were seen between FP and FM (*p* < 0.05). Significant differences in the axial and occlusal areas remained between ZLSMP and ZLSP after cementing and thermocycling.

In vitro, thermocycling showed insignificant differences in the marginal area for all the tested crowns (*p* > 0.05).

## 4. Discussion

The first hypothesis was rejected since the material type and processing method had a significant effect on the values of marginal adaptation. The second hypothesis was confirmed, as thermal aging did not have a significant effect on the marginal adaptation values. 

In this study, different levels of adaptation were calculated (marginal, axial, and occlusal) to gather a complete picture with regard to the crown settings on the resin abutments, before and after cementing and thermocycling.

In vitro studies have the benefit of obtaining results earlier than in a vivo evaluation. This study investigated the adaptation of a typodont maxillar premolar. The results of this study support the idea that different types of ceramic, the processing technology, cementing the restorations, and thermocycling influence the marginal adaptation.

Marginal and internal gap measurements are essential factors in the adaptation of ceramic restorations and lead to clinical success. A perfect fit contributes to gingival health and reduces percolation, which can lead to cement dissolution [39,40,41,42]. Marginal adaptation of all-ceramic restorations can be affected by several factors depending on the selected processing technique of the ceramic materials. 

Clinicians need to take into consideration several aspects when working with a CAD/CAM system. Some of this aspects are the accuracy and precision of the scanning. Dental restorations with great marginal and internal fitness are the results of accurate scanning. Besides the scanning stage, CAD/CAM ceramic restoration can be affected by fabricating technique [43] In the heat-pressing technique, ceramic restorations can encounter changes during the investing and sintering process, which can lead to distortions.

In this study, a standard tooth preparation was multiplied using an additive process to reduce additional errors that can occur in a non-additive way of multiplying the standard tooth preparation. Additionally, the printed resin was elected to mimic the mechanical properties of the natural structures. The milled restorations were obtained from ceramic blocks, and the wax for the heat-pressed technique was also obtained from milling wax blanks following the same design. This protocol was followed to provide standardization and to eliminate the errors than can occur during the processes of obtaining ceramic restorations as much as possible [44].

A chamfer preparation was selected based on similar studies that evaluated the effect of different marginal lines [45]. In other studies, there was no statistical difference between the chamfer lines and shoulder preparation. Clinically, the chamfer preparation provides more advantages compared to the other marginal line [46,47,48]. For the success of CAD/CAM technologies, preparations such as feather-edge with deep retentive grooves and complex occlusal morphology are not indicated for scanning and milling. In some cases, the diameter of the milling bur could be larger than some parts of the crown and this could lead to a larger internal gap compared to the heat-pressed technology.

To assess the internal gap, several methods have been developed including replica technique and micro-CT scanning. Both of these techniques have benefits to preserve the integrity of the samples. The replica technique has the advantages of being reproducible and non-invasive, leaving the evaluated restoration intact [49]. Some drawbacks can also be spotted in this technique: one of them is its difficulty in stabilizing the thin and fragile space between the crown and resin abutment [50]. With the use of this technique, the internal fit can be evaluated at several chosen points along the circumferences of the restoration [51]. In this study, the silicon all remained in the crown and not on the resin abutments. In order to pursue the investigation, putty silicon had to be applied. Another aspect in this stage is the applied force, but studies show that the force does not have a significant impact on the thickness of the light silicon. This replica technique did not provide information regarding the cemented restorations on the abutments, and another non-invasive method, micro CT, was selected to measure the marginal adaptation after cementing the crowns and thermocycling them.

In this study, the results showed that the marginal and occlusal gaps were greater than the axial gap in all the tested groups before and after cementation and thermal aging. This aspect can be explained by the hydraulic pressure (of the light body silicon before, and cement after), which forces the material in the occlusal and marginal areas. Furthermore, this can be related to the axial convergence of the walls, which allows the flowing of the cement material and its continuous escape until reaching the minimum possible thickness [52]. These findings are also similar to other studies [53]. Another published study concluded that the marginal gap had the lowest value, but this may come from the difference in the CAD/CAM scanning system and measurement method [54]. Greater gaps in the occlusal area compared to other areas (marginal and axial) can be explained by the limitation of the scanner resolution, which may produce rounded edges [55]. Another explanation can result from the planar occlusal reduction compared to the flat occlusal reduction design used in other studies as this type of design can produce more occlusal gaps due to scanning. These findings are in agreement with those in previous studies [56].

The best marginal and internal fitness before and after cementing and thermal aging were shown in the FM crowns. This type of crown was not subjected to high temperatures from sintering like the other crowns, and this provides a degree of dimensional accuracy of the final restoration. This aspect has a significant impact on the values of the internal and marginal fitness of crowns [57].

Even though the chemical composition is the same, the processing technologies differ. ZLSM crowns showed a similar marginal adaptation compared to ZLSP, but different internal fitness. This is a result of the processing technology. The ZLSM crowns were subjected to a 25 min crystallization process compared to the ZLSP crowns that were processed for an hour at a temperature of 900 degrees Celsius. The FP crowns showed the highest values for marginal and internal adaptability, and this can be a consequence of multiple firings that take place in the heat-pressed technique. 

The results after cementation provided a good marginal fit for the CAD/CAM restorations, and this could be explained based on their internal relief and smooth surfaces, which allow the luting agents to flow quickly and reduce the marginal discrepancies [58]. An explanation regarding the increased internal gap after luting in both groups (heat-pressed and milled) can be explained by microfractures that can appear as a result of lacking elastic deformation of ceramic materials. After thermocycling, the difference in the marginal area could be explained by the successive temperatures that lead to thermal expansions and can cause a rupture of the bond between the luting agent and the resin abutments [59]. The results of this study show that all the samples were not significantly affected by thermal aging in the marginal area.

Several studies found in literature have evaluated the marginal gap between 100–200 µm and the internal gap from 200–300 µm for the cemented restorations [60,61,62,63]. These values are clinically acceptable. The findings of this study were similar.

An essential aspect that limited the variation of internal adaptation was the cement space, and its thickness, which can be chosen from the design stage. Studies recommend a space of 30 up to 60 µm thick, but further studies have to be carried out.

The differences between the materials included in this study also come from the need for the post-milling processing of the ZSL glass-ceramic. These differences in the processing can have an impact on the marginal and internal adaptation of restoration. Another aspect is the heat-pressing technique that can produce differences in the marginal and internal adaptation.

## 5. Conclusions

Within the limitations of this study, some conclusions can be drawn:

(1) For all the tested samples related to the investigated areas, different levels of adaptation were registered, and the greatest gap was seen in the occlusal, especially for the heat-pressed crowns.
(2) The type of the selected ceramic influenced the marginal adaptability significantly, especially for the FM and ZLSP.
(3) The technological processes influenced the marginal and internal fit of the crowns in favor of the CAD/CAM technologies.
(4) Thermal aging had an insignificant effect on marginal adaptability.
(5) The difference in the chemical composition of the tested materials in this study and the difference in processing reflects the difference in the marginal and internal fitness of fabricated all-ceramic crowns.

## Figures and Tables

**Table 1:** CAD-CAM ceramic materials were selected for this study.

Material	Composition	Manufacturer	Translucency/Shade
1. Vita Mark II FM (milled feldspathic glass-ceramic)	<20 wt.% feldspathic particles (average particle size 4 µm) >80 wt.% glass matrix	Vita Zahnfabrick, Bad Säckingen, Germany	MT/A2
2. Vita Suprinity ZLSM (milled zirconia reinforced lithium silicate glass-ceramic)	The silica content of 55–65 wt.% the lithia (15–21 wt.%) zirconia (8–12 wt.%) nanoparticle size 0.5–0.7 µm	Vita Zahnfabrick, Bad Säckingen, Germany	MT/A2

**Table 2:** Heat-pressed ceramic materials included in this study.

Material	Composition	Manufacturer	Translucency/Shade
1. Vita PM9 FP (heat-pressed feldspathic glass-ceramic)	50% of Leucite reinforced glass-ceramic (size 10–15 µm) s	Vita Zahnfabrick, Bad Säckingen, Germany	MT/A2
2. Celtra Press ZLSP (zirconia reinforced lithium silicate glass-ceramic)	A glass matrix and lithium disilicate crystals 1.5 µm plus nanoscale lithium 10% (ZrO_2_)	Dentsply, Hanau, Germany	MT/A2

**Table 3:** Type of glaze for each ceramic.

Type of Ceramic	Type of Glaze
FP, FM, ZLSM	Vita Akzent Plus Glaze LT (Vita Zahnfabrick, Bad Säckingen, Germany)
ZLSP	Dentsply Universal stain (Dentsply, Hanau, Germany)

**Table 4:** Mean values (µm) and standard deviations (SD) of the tested materials before cementing and thermocycling.

Ceramic Material	Marginal Mean (µm)	Axial Mean (µm)	Occlusal Mean (µm)
FP	90.5 ± 43.36	66.5 ± 36.72	132.5 ± 82.20
FM	65.2 ± 27.78	60.5 ± 34.32	93.6 ± 52.61
ZLSP	86.7 ± 38.95	84.2 ± 46.38	131.6 ± 86.35
ZLSM	81.3 ± 39.09	64.2 ± 34.85	119.2 ± 81.48

**Table 5:** Statistical results before cementing and thermocycling the samples.

*p*-Value (*p* < 0.05)			
Ceramic Material	Marginal Mean	Axial Mean	Occlusal Mean
FP-FM	0.022	0.4	0.08
ZLSP-ZLSM	0.8	0.028	0.0328
FP-ZLSP	0.053	0.47	0.453
FP-ZLSM	0.05	0.3	0.048
FM-ZLSP	0.02	0.034	0.015
FM-ZLSM	0.06	0.458	0.562

**Table 6:** Mean values (µm) and standard deviations (SD) of the tested materials after cementing and thermocycling.

Ceramic Material	Marginal Mean (µm)	Axial Mean (µm)	Occlusal Mean (µm)
FP	96.2 ± 44.54	76.1 ± 50.38	135.5 ± 92.58
FM	71.3 ± 30.43	61.5 ± 35.69	96.6 ± 54.42
ZLSP	91.6 ± 41.22	86.2 ± 48.50	135.9 ± 87.02
ZLSM	85.2 ± 39.97	65.5 ± 36.74	121.2 ± 92.43

**Table 7:** Statistical analysis after cementing and thermocycling the samples.

*p*-Value (*p* < 0.05)			
Ceramic Material	Marginal Mean	Axial Mean	Occlusal Mean
FP-FM	0.02	0.157	0.134
ZLSP-ZLSM	0.9	0.023	0.035
FP-ZLSP	0.256	0.407	0.458
FP-ZLSM	0.123	0.07	0.043
FM-ZLSP	0.05	0.107	0.108
FM-ZLSM	0.04	0.33	0.166
FP-t-FP	0.5	0.38	0.385
FM-t-FM	0.2	0.124	0.465
ZLSP-t-ZLSP	0.06	0.2	0.468
ZLSM-t-ZLSM	0.5	0.4	0.122

t—Thermocycling.
